# Use High-Flow Nasal Cannula for Acute Respiratory Failure Patients in the Emergency Department: A Meta-Analysis Study

**DOI:** 10.1155/2019/2130935

**Published:** 2019-10-13

**Authors:** Cheng-Chieh Huang, Hao-Min Lan, Chao-Jui Li, Tsung-Han Lee, Wen-Liang Chen, Wei-Yuan Lei, Pei-You Hsieh, Mei-Chueh Yang, Chu-Chung Chou, Han-Ping Wu, Yuan-Jhen Syue

**Affiliations:** ^1^Department of Emergency Medicine, Changhua Christian Hospital, Changhua, Taiwan; ^2^Department of Biological Science and Technology, National Chiao Tung University, Hsinchu, Taiwan; ^3^Department of Education, Kaohsiung Chang Gung Memorial Hospital, Chang Gung University College of Medicine, Taoyuan, Taiwan; ^4^Department of Emergency Medicine, Kaohsiung Chang Gung Memorial Hospital, Chang Gung University College of Medicine, Kaohsiung, Taiwan; ^5^Department of Leisure and Sports Management, Cheng Shiu University, Kaohsiung, Taiwan; ^6^School of Medicine, Kaohsiung Medical University, Kaohsiung, Taiwan; ^7^School of Medicine, Chung Shan Medical University, Taichung, Taiwan; ^8^Department of Pediatric Emergency Medicine, Children Hospital, China Medical University, Taichung, Taiwan; ^9^Department of Medicine, School of Medicine, China Medical University, Taichung, Taiwan; ^10^Department of Medical Research, Children's Hospital, China Medical University, Taichung, Taiwan; ^11^Department of Anaesthesiology, Kaohsiung Chang Gung Memorial Hospital, Chang Gung University College of Medicine, Kaohsiung, Taiwan

## Abstract

**Objective:**

To evaluate the efficacy of high-flow nasal cannula (HFNC) therapy compared with conventional oxygen therapy (COT) or noninvasive ventilation (NIV) for the treatment of acute respiratory failure (ARF) in emergency departments (EDs).

**Method:**

We comprehensively searched 3 databases (PubMed, EMBASE, and the Cochrane Library) for articles published from database inception to 12 July 2019. This study included only randomized controlled trials (RCTs) that were conducted in EDs and compared HFNC therapy with COT or NIV. The primary outcome was the intubation rate. The secondary outcomes were the mortality rate, intensive care unit (ICU) admission rate, ED discharge rate, need for escalation, length of ED stay, length of hospital stay, and patient dyspnea and comfort scores.

**Result:**

Five RCTs (*n* = 775) were included. There was a decreasing trend regarding the application of HFNC therapy and the intubation rate, but the difference was not statistically significant (RR, 0.53; 95% CI, 0.26–1.09; *p*=0.08; *I*^2^ = 0%). We found that compared with patients who underwent COT, those who underwent HFNC therapy had a reduced need for escalation (RR, 0.41; 95% CI, 0.22–0.78; *p*=0.006; *I*^2^ = 0%), reduced dyspnea scores (MD −0.82, 95% CI −1.45 to −0.18), and improved comfort (SMD −0.76 SD, 95% CI −1.01 to −0.51). Compared with the COT group, the HFNC therapy group had a similar mortality rate (RR, 1.25; 95% CI, 0.79–1.99; *p*=0.34; *I*^2^ = 0%), ICU admission rate (RR, 1.11; 95% CI, 0.58–2.12; *p*=0.76; *I*^2^ = 0%), ED discharge rate (RR, 1.04; 95% CI, 0.63–1.72; *p*=0.87; *I*^2^ = 0%), length of ED stay (MD 1.66, 95% CI −0.95 to 4.27), and hospital stay (MD 0.9, 95% CI −2.06 to 3.87).

**Conclusion:**

Administering HFNC therapy in ARF patients in EDs might decrease the intubation rate compared with COT. In addition, it can decrease the need for escalation, decrease the patient's dyspnea level, and increase the patient's comfort level compared with COT.

## 1. Background

Acute respiratory failure (ARF) is a critical condition faced in emergency departments (EDs). It can result from many conditions, such as cardiogenic pulmonary edema, pneumonia, or acute exacerbation of chronic obstructive pulmonary disease and has a high mortality rate [1]. Conventional oxygen therapy (COT), including a nasal cannula, face mask, venturi mask, and nonrebreathing mask, can be provided to correct hypoxemia. However, the maximal flow rate of COT devices is 15 L/min, which is not enough for patients with ARF. Thus, escalating oxygen therapy to noninvasive ventilation (NIV, e.g., biphasic positive airway pressure) or invasive ventilation may be needed.

Some studies have demonstrated that intubation in ARF patients is associated with an increased complication rate and mortality rate when compared with NIV [2–4]. Even so, NIV is associated with some disadvantages, such as gastric distension, vomiting, claustrophobia, possible nasal skin damage, and difficulty in speaking and coughing, and may lead to treatment failure [5]. According to a previous report, the NIV failure rate in ARF patients ranges from 5% to 60%, depending on numerous factors [6]. Another investigation revealed that up to 25% of chronic obstructive pulmonary disease acute exacerbation patients do not tolerate NIV for several reasons [7]. Therefore, using an ideal NIV device for patients not only improves comfort and dyspnea levels but also decreases intubation and mortality rates potentially.

In recent years, many studies have shown clinical benefits associated with high-flow nasal cannula (HFNC) therapy in ARF patients [8], the oxygen support of preoxygenation [9], acute pulmonary edema [10], the maintenance of oxygenation during bronchoscopy [11], and the prevention of reintubation [12] because an HFNC can provide warmed, humidified, and up to 100% oxygen. When compared to COT and NIV, HFNC therapy has some potential advantages. First, an HFNC can deliver a constant and wide FiO_2_ range according to the patient's needs. Second, a maximum flow of 60 L/min can generate positive end-expiratory pressure, resulting in the elimination of some airway dead space, improving oxygenation [13]. Third, inspired warm and humidified oxygen can optimize mucosal functions, maximize mucociliary clearance and help expectoration [14]. Finally, using an HFNC can decrease the interruption of oxygen therapy (e.g., during eating, drinking, or talking) and increase patient compliance, resulting in potentially improved outcomes [15]. Previous systematic reviews analyzing heterogeneous study methods (combining observational and randomized controlled trial (RCT) data [16]) and populations (combined ICU and ED populations [17]; those with ARF, and postextubation and postoperation populations [18, 19]) may cause controversial results. Thus, clarifying the use of HFNCs for ARF patients in EDs is necessary. In this study, we conducted a recent systematic review and meta-analysis to evaluate the differences between using HFNC therapy and COT or NIV in ARF patients in EDs.

## 2. Methods

This study design followed the Cochrane Handbook for Systematic Reviews of Interventions guidelines [20] and Preferred Reporting Items for Systematic Reviews and Meta-Analyses (PRISMA) statements [21].

## 3. Eligibility Criteria

### 3.1. Types of Studies

Only RCTs were eligible. We excluded retrospective studies, observational studies, before-after studies, crossover studies, case reports, abstract publications, and conference presentations.

### 3.2. Types of Participants

We included adult patients (>18 years old) with ARF due to any cause admitted to ED. “ARF” was defined as an SpO2 <92% in room air, a PaO_2_/FiO_2_ (ratio of the partial pressure of arterial oxygen to the fraction of inspired oxygen) <300, clinical symptoms and signs (including a respiratory rate >24 breaths per minute, the use of accessory muscles to breath, or shortness of breath at rest) or “author” definitions. Postoperation, postextubation, and ICU patients were all excluded.

### 3.3. Types of Interventions

Studies comparing HFNC therapy with COT and/or other NIV devices were included. There was no oxygen flow or concentration restriction for the intervention group (HFNC) or the comparison group (COT and/or other NIV devices).

### 3.4. Types of Outcome Measures

Our primary outcome was the intubation rates of both of the groups. The secondary outcomes were the mortality rate, ICU admission rate, ED discharge rate, need for escalation, length of ED stay, length of hospital stay, and patient dyspnea and comfort scores. We also considered 2 subgroup analyses according to the intervention device (HFNC versus COT and NIV versus COT) and treatment duration (HFNC ≤2 hours versus COT and HFNC >2 hours versus COT).

### 3.5. Search Methods for Identification of Studies

We comprehensively searched 3 databases (PubMed, EMBASE, and the Cochrane Library) for articles published from database inception to 12 July 2019. The following key words or medical subject headings (MeSH) terms were used: *high-flow nasal cannula*, *high-flow nasal*, *high-flow oxygen therapy*, or *high-flow therapy*, and *emergency department*, *emergency room*, *emergency unit*, *or emergency service*. To avoid the loss of possible studies, we also reviewed the references of the identified articles. No language restriction was applied.

### 3.6. Data Extraction, Quality Assessment, and Grading of the Quality of Evidence

Two authors (CCH and HML) extracted the data from the reviewed articles independently. We used an unweighted kappa score to test interrater reliability. If any disagreement occurred, it was resolved by discussion, consensus, or consultation with a third author (CJL). The following data were collected for each eligible study: authors, publication year, study design, study group, intervention/control detail, and outcome data.

The risk of bias was independently assessed by two authors (CCH and HML) according to the Cochrane Handbook for Systematic Reviews of Interventions guidelines, chapter 8 [20]. There were 7 domains that were assessed for each study: random sequence generation, allocation concealment, blinding of participants and personnel, blinding of outcome assessment, incomplete outcome data, selective reporting, and other bias. Each domain was rated as having low risk (green), unclear risk (yellow), or high risk (red).

We also used the Grading of Recommendation, Assessment, Development, and Evaluation (GRADE) method [22] to evaluate the quality of evidence, which was classified as very low, low, moderate, or high, for the primary and secondary outcomes.

### 3.7. Statistical Analysis

All data were analyzed by Review Manager (RevMan, Version 5.3, Copenhagen: The Nordic Cochrane Center, The Cochrane Collaboration, 2014). Because some of the included studies presented results as medians, interquartile ranges, or minimum/maximum values, we use the Wan et al. method to estimate the sample mean and standard deviation [23]. We expressed dichotomous data as risk ratios (RRs) and continuous data as mean differences (MDs) or standardized mean differences (SMDs). For all of the results, 95% confidence intervals (CIs) were calculated. To evaluate heterogeneity, we used chi-square and *I*^2^ tests. If the heterogeneity was nonsignificant (*I*^2^ < 50%), we applied fixed-effects models; otherwise, we applied random-effects models for analysis. In addition, we performed a visual inspection of the funnel plot to assess publication bias, and a sensitivity analysis was conducted by repeating the analysis after removing one RCT at a time. Finally, the results were presented in forest plots.

## 4. Result

**Figure 1:**
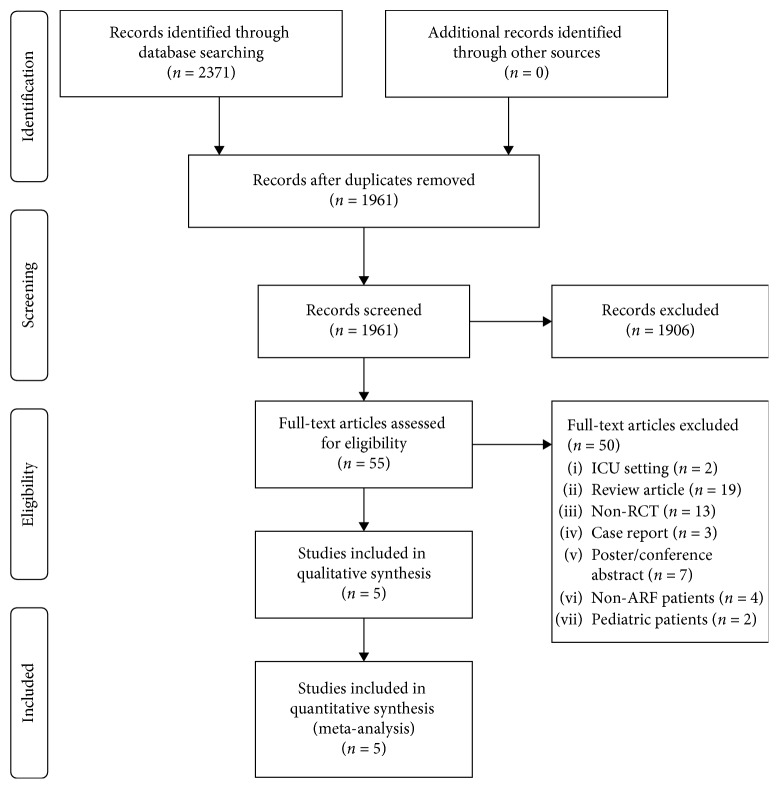
PRISMA flow diagram.

A total of 2371 potentially eligible studies were initially identified. After screening titles and abstracts, 55 full-text studies were retrieved for eligibility screening. Then, we excluded studies conducted in ICU settings, review articles, nonrandomized controlled trials, case reports, conference abstracts, studies involving non-ARF patients, and studies involving pediatric patients. Finally, 5 RCTs [24–28] including 775 patients were entered into our meta-analysis (Figure 1). The interrater reliability of study screening (kappa score, 0.76; 95% CI, 0.68–0.86) and risk of bias assessment (kappa statistic 0.77, 95% CI: 0.57–0.98) were good. The mean age ranged from 63.4 to 73.7 years old. Of the 5 RCTs, 1 RCT [28] compared HFNC therapy with NIV and the others [24–27] compared HFNC therapy with COT (nasal cannula, face mask, venturi mask, or nonrebreathing mask). The flow in the HFNC group was 35 L/min or more at initiation, and the duration of therapy ranged from 1 hour to 72 hours. The main causes of ARF were chronic obstructive pulmonary disease (COPD), pneumonia, and cardiac-related disease in our included studies. The basic characteristics of the included studies are shown in Table 1. Owing to the lack of an NIV group in the other studies, we were not able to analyze the RCT [28] that compared HFNC with NIV. In addition, there was no event (intubation) in one RCT [25], and the data in the included studies were too insufficient to obtain valuable conclusions. Because of the above 2 reasons, we were not able to perform subgroup analyses.

### 4.1. Risk of Bias of the Included Studies

**Figure 2:**
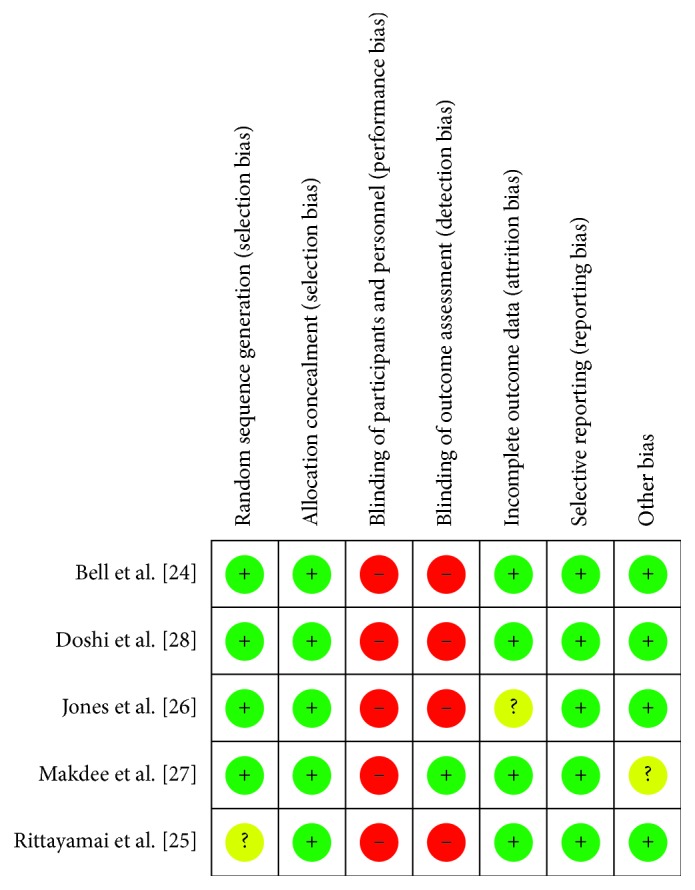
Risk of bias summary.

The risk of selection bias in our included studies was all low, except for 1 RCT [25] that had an unclear risk of bias. The risk of performance bias was all high because it was impossible to blind patients and personnel in the clinical setting when comparing HFNC to COT. The other risk of bias results is shown in Figure 2.

### 4.2. Quality Assessment

Table 2 summarizes all outcomes and the quality of evidence of the articles included in this meta-analysis. The intubation rate, length of ED/hospital stay, and patient dyspnea level were of low quality, and the others were of moderate quality. A visual inspection of the funnel plot revealed no publication bias (Additional [Supplementary-material supplementary-material-1]).

### 4.3. Primary Outcome

**Figure 3:**
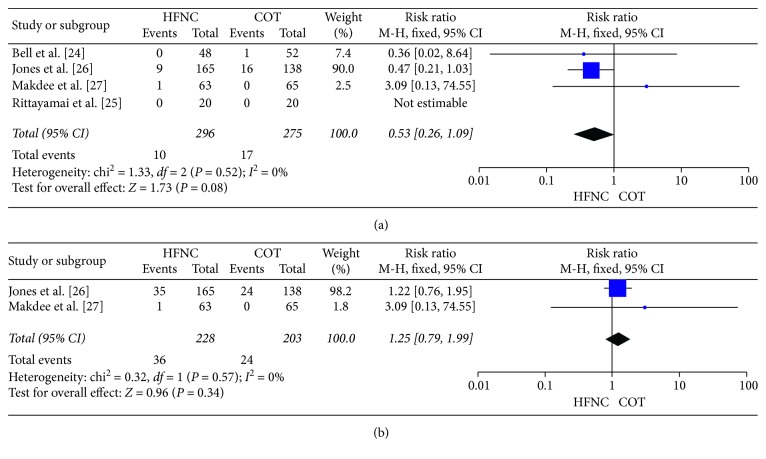
Intubation rate and mortality rate. (a) Intubation rate: HFNC group versus COT group. (b) Mortality rate: HFNC group versus COT group.

Four RCTs including 571 patients reported the intubation rates for both groups. Ten of 296 (3.38%) patients in the HFNC group were intubated, and 17 of 275 (6.18%) patients in the COT group were intubated. There was a decreasing trend of HFNC therapy and intubation rate, but it was not statistically significant (RR, 0.53; 95% CI, 0.26–1.09; *p*=0.08; *I*^2^ = 0%) (Figure 3(a)).

### 4.4. Secondary Outcomes

#### 4.4.1. Mortality, ICU Admission Rate, and ED Discharge Rate

**Figure 4:**
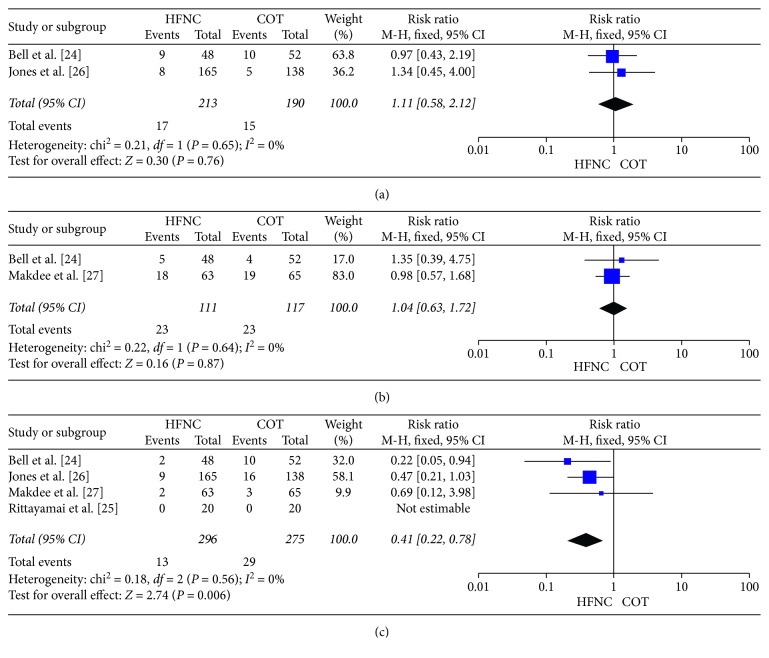
Other secondary outcomes. (a) ICU admission rate: HFNC group versus COT group. (b) ED discharge rate: HFNC group versus COT group. (c) Need for escalation: HFNC group versus COT group.

We did not observe a difference in the mortality rate (RR, 1.25; 95% CI, 0.79–1.99; *p*=0.34; *I*^2^ = 0%), ICU admission rate (RR, 1.11; 95% CI, 0.58–2.12; *p*=0.76; *I*^2^ = 0%), or ED discharge rate (RR, 1.04; 95% CI, 0.63–1.72; *p*=0.87; *I*^2^ = 0%) between the HFNC and COT groups (Figures 3(b), 4(a), and 4(b)).

#### 4.4.2. Need for Escalation

If the patient could not tolerate initial therapy (HFNC therapy or COT) or therapy failed, patient oxygenation required escalation to avoid hypoxia. The escalation strategies in our included studies were similar. Two RCTs [26, 27] escalated to NIV or invasive ventilation in both the HFNC and COT groups. One RCT [24] escalated to NIV or invasive ventilation in the HFNC group and HFNC, NIV or invasive ventilation in the COT group. No escalation was needed in 1 RCT [25]. HFNC therapy decreased the need for escalation compared to COT (RR, 0.41; 95% CI, 0.22–0.78; *p*=0.006; *I*^2^ = 0%) (Figure 4(c)).

#### 4.4.3. Length of ED Stay and Hospital Stay

The length of ED stay (MD 1.66, 95% CI −0.95 to 4.27) and hospital stay (MD 0.9, 95% CI −2.06 to 3.87) were similar in both the HFNC and COT groups (Table 2).

#### 4.4.4. Dyspnea Score

The dyspnea score in the HFNC group was significantly lower than that in the COT group (MD −0.82, 95% CI −1.45 to −0.18) (Table 2). Two of the 4 RCTs reported measurable dyspnea scores. One RCT [25] used a numerical rating scale ranging from 0 to 10, and the other RCT [27] used a visual analog scale ranging from 0 to 10. Of the included RCTs, 1 RCT [24] defined patient dyspnea as a reduction in the respiratory rate >20% from baseline and a reduction in the Borg score. Both arms showed a significant decrease in the dyspnea level in the HFNC group (reduction in the respiratory rate >20% from baseline: HFNC 32/48 (66.7%), COT 20/52 (38.5%), *p*=0.005; reduction in the Borg score: HFNC 36/48 (75%), COT 29/52 (55.8%), *p*=0.044).

#### 4.4.5. Comfort Score

Because different scoring systems, including a 5-point Likert scale, numerical rating scale, and visual analog scale, were used in different RCTs, we calculated SMDs to evaluate patient comfort levels. Three of the 4 RCTs reported measurable comfort scores, and patients in the HFNC group were more comfortable than those in the COT group (SMD −0.76 SD, 95% CI −1.01 to −0.51) (Table 2).

## 5. Discussion

The most important result of this meta-analysis is that HFNC therapy for ARF patients in EDs can reduce the need for escalation oxygen therapy compared with COT. This result was similar to that in recent meta-analyses [17, 29, 30]. Although there were no differences found in the mortality rate, there was a decreasing trend between HFNC therapy and intubation rate in ARF patients, despite the lack of statistical significance. On the other hand, we did not observe an influence of HFNC therapy on the ICU admission rate, ED discharge rate or length of ED/hospital stay. A meta-analysis by Maitra et al. [31], which included 5 trials (*n* = 759), revealed no difference in the requirement of increased respiratory support between the HFNC therapy and COT groups. However, the study enrolled not only ARF patients but also postcardiac operation patients, and the heterogeneity was high. In addition, Rochwerg et al. [17], Monro–Somerville et al. [19], Bocchile et al. [30] who conducted 3 previous meta-analyses including 7 trials (*n* = 1647), 8 trials (*n* = 1567), and 6 trials (*n* = 839), respectively, demonstrated that HFNC therapy significantly reduced the intubation rate compared to COT. In our study, we did not observe an apparently reduced intubation rate in the HFNC group, probably because of the low patient number.

The other important result of this meta-analysis is that treating ARF patients with HFNC therapy resulted in lower patient dyspnea scores and higher patient comfortable than treating ARF patients with COT. All of our included RCTs revealed that HFNC therapy was better than COT regarding dyspnea and comfort scores, except for 1 RCT [26]. This RCT by Jones et al., which used a 5-point Likert scale to evaluate patient dyspnea and comfort levels, present the results by combining the best 2/other 3 or worst 2/other 3 categories for positive and negative questions, respectively, so we were not able to quantitatively pool these data. HFNC therapy can provide warmed, humidified, and 100% oxygen, which may explain the reduction in the need for escalation oxygen therapy compared to COT. Other features such as removing airway dead space, improving oxygenation, optimizing mucosal functions, maximizing mucociliary clearance, promoting expectoration, and decreasing interruptions to oxygen therapy are also possible reasons for the decreasing trend in the intubation rate, improved patient dyspnea scores, and improved comfort levels associated with HFNC therapy.

In this study, we noted that some targeted studies are heterogeneous with their methods and case mix. For example, three RCTs [24–26] included all kinds of ARF patients (COPD, pneumonia, cardiac-related disease, and others) while 1 RCT [27] only included cardiogenic pulmonary edema patients. In addition, initial FiO_2_ (ranged from 28% to 100%), flow rate (ranged from 35 L/min to 50 L/min) of HFNC, duration of therapy (ranged from 1 hour to 9.3 hours), and authors' definitions of ARF were all different. Although the inclusion studies were heterogeneous, most of the outcomes did not reveal statistical heterogeneity (*I*_2_ or inconsistency). The FiO_2_ and the flow rate needed to titrate to clinical demand in all including patients. Moreover, most improvements in respiratory effort and oxygenation were already obtained at the flow rate of 30 L/min [32]. These are possible reasons to explain consistency of most outcomes despite different cause of ARF and initial settings.

HFNC also plays an important role in acute exacerbations of chronic obstructive pulmonary disease (AECOPD). AECOPD with respiratory failure, a kind of type II respiratory failure, is caused by airflow obstruction or increasing dead space. As previously mentioned, HFNC can generate about 2–4 cm H_2_O positive end-expiratory pressure [33], resulting in decreasing PaCO2 level and improving oxygenation by elimination of some airway dead space [13]. Furthermore, titrated oxygen therapy with target saturation of 88–92% can significantly reduce mortality, hypercapnia, and respiratory acidosis in AECOPD [34]. HFNC is able to deliver a constant and wide FiO_2_ range oxygen, so it can titrate with target saturation of 88–92% depending on clinical needs. According to the 2019 Global Initiative for Chronic Obstructive Lung Disease (GOLD) guideline, HFNC may be an alternative to standard oxygen therapy or noninvasive positive pressure ventilation in AECOPD [35]. There are also some investigations demonstrate using HFNC in COPD patients can decrease PaCO2 level [36], respiratory effort, and improving oxygenation [37]. Thus, applying HFNC to AECOPD patients is reasonable respiratory support.

There was a decreasing trend between HFNC therapy and intubation rate in ARF patients, but there were no differences found in the mortality rate. There was a concern about that delay intubation could increase mortality in ARF patients treating with HFNC. Because HFNC could improve respiratory effort and patients “looked” better initially, they would not be intubated in a timely manner. Kang et al. illustrated failure of HFNC therapy may delay intubation and increase mortality [38]. However, the “delay” was defined as >48 hours after HFNC therapy, which was far from our included RCTs (ranged from 1 hour to 9.3 hours). In addition, there were various etiologies of ARF, such as COPD, pneumonia, pulmonary edema, asthma, pulmonary embolism, and other causes, in the included RCTs. Not every disease initially benefited equally from HFNC therapy. Messika et al. demonstrated that increased breathing frequency, an increased Simplified Acute Physiology Score (SAPS) II score, and decreased PaO_2_/FiO_2_ were associated with HFNC treatment failure [39].

To the best of our knowledge, this is the first meta-analysis regarding the use of HFNC for ARF patients in EDs. The advantages of this analysis include performing a comprehensive article search in 3 databases (PubMed, EMBASE, and the Cochrane Library). In addition, only RCTs involving HFNC therapy, COT or NIV for de novo acute hypoxemic respiratory failure patients in EDs were included in our meta-analysis. Moreover, we used the GRADE method to evaluate the quality of evidence for primary and secondary outcomes. There were also several limitations to this study. Firstly, the number of RCTs comparing HFNC to COT or NIV for patients with ARF in EDs were too few to include in our meta-analysis, so we were not able to perform subgroup analyses, and the low patient number increased the risk of bias. Secondly, our quality of evidence for outcomes was low to moderate and was affected by serious risk of bias, inconsistencies, and imprecision. Finally, we did not have enough power to evaluate publication bias by inspection of a funnel plot because of the small sample size. Therefore, further large-sample studies are warranted to clarify the role of HFNC therapy for ARF patients in EDs.

## 6. Conclusion

In this meta-analysis, we demonstrated that HFNC therapy for ARF patients in the ED might decrease the intubation rate compared to COT. In addition, it can decrease the need for escalation, decrease the patient's dyspnea score, and increase the patient's comfort level compared to COT. Further high-quality and large-sample studies are warranted to confirm the role of HFNC therapy for ARF patients in EDs.

## Figures and Tables

**Table 1:** The basic characteristics of the included studies.

Study, year	Design	Setting	Age, years^*∗*^	Cause of ARF	Sample size			Duration of therapy
					HFNC	COT	NIV	
Bell et al. [24]	RCT	ED	73.7 ± 17.5	COPD, respiratory tract infection, cardiac related, pulmonary embolism, asthma	48	52	—	2 h
Rittayamai et al. [25]	RCT	ED	64.6 ± 15.1	CHF, pneumonia, asthma, COPD, others	20	20	—	1 h
Jones et al. [26]	RCT	ED	73.5 ± 16.2	COPD, pneumonia, asthma, others	165	138	—	5 h
Makdee et al. [27]	RCT	ED	70 ± 15	Cardiogenic pulmonary edema	63	65	—	2.9 h (0.2–9.3 h)
Doshi et al. [28]	RCT	ED	63.4 ± 14	COPD, CHF, pneumonia, asthma	104	—	100	72 h

RCT, randomized controlled trial; ED, emergency department; COPD, chronic obstructive pulmonary disease; CHF, congestive heart failure; h, hours. ^*∗*^Mean ± standard deviation (SD).

**Table 2:** The summary of outcomes and GRADE quality assessments.

Quality assessment							No of patients		Effect		Quality	Importance
No. of studies	Design	Risk of bias	Inconsistency	Indirectness	Imprecision	Other consideration	HFNC	COT	Relative RR (95% CI)	Absolute (95% CI)		
Intubation rate												
4	RCTs	Serious^a^	Not serious	Not serious	Serious^b^	None	10/296 (3.4%)	17/275 (6.2%)	0.53(0.26–1.09)	−32 per 1000 (from −69 to 4)	⊕⊕Low	Critical

Mortality rate												
2	RCTs	Not serious	Not serious	Not serious	Serious^b^	None	36/228 (15.8%)	24/203 (11.8%)	1.25(0.79–1.99)	32 per 1000 (from −32 to 95)	⊕⊕⊕Moderate	Critical

ICU admission rate												
2	RCTs	Not serious	Not serious	Not serious	Serious^b^	None	17/213 (8%)	15/190 (7.9%)	1.11(0.58–2.12)	8 per 1000 (from −43 to 59)	⊕⊕⊕Moderate	Critical

ED discharge rate												
2	RCTs	Not serious	Not serious	Not serious	Serious^b^	None	23/111 (20.7%)	23/117 (19.7%)	1.04(0.63–1.72)	8 per 1000 (from −93 to 109)	⊕⊕⊕Moderate	Critical

Need for escalation												
4	RCTs	Serious^a^	Not serious	Not serious	Not serious	None	13/296 (4.4%)	29/275 (10.5%)	0.41(0.22–0.78)	−62 per 1000 (from −105 to −19)	⊕⊕⊕Moderate	Critical

Length of ED stay (hour)												
3	RCTs	Not serious	Serious^c^	Not serious	Serious^b^	None	276	255	—	MD 1.66 hours (from −0.95 to 4.27)	⊕⊕Low	Critical

Length of hospital stay (day)												
2	RCTs	Not serious	Serious^c^	Not serious	Serious^b^	None	228	203	—	MD 0.9 days (from −2.06 to 3.87)	⊕⊕Low	Critical

Patient dyspnea score												
2	RCTs	Serious^d^	Serious^c^	Not serious	Not serious	None	83	85	—	MD −0.82 point (from −1.45 to −0.18)	⊕⊕Low	Critical

Patient comfort score												
3	RCTs	Serious^d^	Not serious	Not serious	Not serious	None	131	137	—	SMD −0.76 SD (from −1.01 to −0.51)	⊕⊕⊕Moderate	Critical

RCT, randomized controlled trial; HFNC, high-flow nasal cannula; COT, conventional oxygen therapy; CI, confidence interval; RR, risk ratio; MD, mean difference; SMD, standardized mean difference. ^a^All inclusion trials lacked blinding (performance bias), so escalation or intubation may be subjective. ^b^Insufficient evidence of clear benefit or harm because of a wide CI. ^c^Significant heterogeneity among the included trials (*I*^2^ > 50%). ^d^Subjective outcome. ⊕, very low quality; ⊕⊕, low quality; ⊕⊕⊕, moderate quality; ⊕⊕⊕⊕, high quality.

## Data Availability

All data are available for all users. We comprehensively searched 3 databases (PubMed, EMBASE, and the Cochrane Library) for articles published from database inception to 12 July 2019. Only randomized controlled trials (RCTs) were conducted in EDs and HFNC therapy was compared to COT or NIV.

## Supplementary Materials

Supplementary Materials A visual inspection of the funnel plot revealed no publication bias.

## References

[1] Ray P., Birolleau S., Lefort Y. (2006). Acute respiratory failure in the elderly: etiology, emergency diagnosis and prognosis. *Critical Care*.

[2] Antonelli M., Conti G., Rocco M. (1998). A comparison of noninvasive positive-pressure ventilation and conventional mechanical ventilation in patients with acute respiratory failure. *New England Journal of Medicine*.

[3] Azevedo L. C. P., Caruso P., Silva U. V. A. (2014). Outcomes for patients with cancer admitted to the ICU requiring ventilatory support. *Chest*.

[4] Confalonieri M., Potena A., Carbone G., Porta R. D., Tolley E. A., Umberto Meduri G. (1999). Acute respiratory failure in patients with severe community-acquired pneumonia. *American Journal of Respiratory and Critical Care Medicine*.

[5] Nava S., Hill N. (2009). Non-invasive ventilation in acute respiratory failure. *The Lancet*.

[6] Ozyilmaz E., Ugurlu A. O., Nava S. (2014). Timing of non-invasive ventilation failure: causes, risk factors, and potential remedies. *BMC Pulmonary Medicine*.

[7] Confalonieri M., Garuti G., Cattaruzza M. S. (2005). A chart of failure risk for noninvasive ventilation in patients with COPD exacerbation. *European Respiratory Journal*.

[8] Frat J. P., Thille A. W., Mercat A. (2015). High-flow oxygen through nasal cannula in acute hypoxemic respiratory failure. *New England Journal of Medicine*.

[9] Doyle A. J., Stolady D., Mariyaselvam M. (2016). Preoxygenation and apneic oxygenation using transnasal humidified rapid-insufflation ventilatory exchange for emergency intubation. *Journal of Critical Care*.

[10] Haywood S. T., Whittle J. S., Volakis L. I. (2019). HVNI vs. NIPPV in the treatment of acute decompensated heart failure: subgroup analysis of a multi-center trial in the ED. *American Journal of Emergency Medicine*.

[11] Chung S. M., Choi J. W., Lee Y. S. (2019). Clinical effectiveness of high-flow nasal cannula in hypoxaemic patients during bronchoscopic procedures. *Tuberculosis and Respiratory Diseases*.

[12] Hernández G., Vaquero C., Colinas L. (2016). Effect of postextubation high-flow nasal cannula vs. noninvasive ventilation on reintubation and postextubation respiratory failure in high-risk patients. *JAMA*.

[13] El-Khatib M. F. (2012). High-flow nasal cannula oxygen therapy during hypoxemic respiratory failure. *Respiratory Care*.

[14] Spoletini G., Alotaibi M., Blasi F., Hill N. S. (2015). Heated humidified high-flow nasal oxygen in adults. *Chest*.

[15] Frat J. P., Brugiere B., Ragot S. (2015). Sequential application of oxygen therapy via high-flow nasal cannula and noninvasive ventilation in acute respiratory failure: an observational pilot study. *Respiratory Care*.

[16] Ni Y. N., Luo J., Yu H. (2017). Can high-flow nasal cannula reduce the rate of endotracheal intubation in adult patients with acute respiratory failure compared with conventional oxygen therapy and noninvasive positive pressure ventilation?. *Chest*.

[17] Rochwerg B., Granton D., Wang D. X. (2019). High flow nasal cannula compared with conventional oxygen therapy for acute hypoxemic respiratory failure: a systematic review and meta-analysis. *Intensive Care Medicine*.

[18] Nedel W. L., Deutschendorf C., Moraes Rodrigues Filho E. (2017). High-flow nasal cannula in critically ill subjects with or at risk for respiratory failure: a systematic review and meta-analysis. *Respiratory Care*.

[19] Monro-Somerville T., Sim M., Ruddy J., Vilas M., Gillies M. A. (2017). The effect of high-flow nasal cannula oxygen therapy on mortality and intubation rate in acute respiratory failure. *Critical Care Medicine*.

[20] Higgins J. P. T., Green S. (2011). Preparing a cochrane review. *Cochrane Handbook for Systematic Reviews of Interventions Version 5.1.0*.

[21] Moher D., Liberati A., Tetzlaff J., Altman D. G. (2009). Preferred reporting items for systematic reviews and meta-analyses: the PRISMA statement. *Annals of Internal Medicine*.

[22] Guyatt G. H., Oxman A. D., Vist G. E. (2008). GRADE: an emerging consensus on rating quality of evidence and strength of recommendations. *BMJ*.

[23] Wan X., Wang W., Liu J., Tong T. (2014). Estimating the sample mean and standard deviation from the sample size, median, range and/or interquartile range. *BMC Medical Research Methodology*.

[24] Bell N., Hutchinson C. L., Green T. C., Rogan E., Bein K. J., Dinh M. M. (2015). Randomised control trial of humidified high flow nasal cannulaeversusstandard oxygen in the emergency department. *Emergency Medicine Australasia*.

[25] Rittayamai N., Tscheikuna J., Praphruetkit N., Kijpinyochai S. (2015). Use of high-flow nasal cannula for acute dyspnea and hypoxemia in the emergency department. *Respiratory Care*.

[26] Jones P. G., Kamona S., Doran O., Sawtell F., Wilsher M. (2016). Randomized controlled trial of humidified high-flow nasal oxygen for acute respiratory distress in the emergency department: the HOT-ER study. *Respiratory Care*.

[27] Makdee O., Monsomboon A., Surabenjawong U. (2017). High-flow nasal cannula versus conventional oxygen therapy in emergency department patients with cardiogenic pulmonary edema: a randomized controlled trial. *Annals of Emergency Medicine*.

[28] Doshi P., Whittle J. S., Bublewicz M. (2018). High-velocity nasal insufflation in the treatment of respiratory failure: a randomized clinical trial. *Annals of Emergency Medicine*.

[29] Xu Z., Li Y., Zhou J. (2018). High-flow nasal cannula in adults with acute respiratory failure and after extubation: a systematic review and meta-analysis. *Respiratory Research*.

[30] Bocchile R. L. R., Cazati D. C., Timenetsky K. T., Neto A. S. (2018). The effects of high-flow nasal cannula on intubation and re-intubation in critically ill patients: a systematic review, meta-analysis and trial sequential analysis. *Revista Brasileira de Terapia Intensiva*.

[31] Maitra S., Som A., Bhattacharjee S., Arora M. K., Baidya D. K. (2016). Comparison of high-flow nasal oxygen therapy with conventional oxygen therapy and noninvasive ventilation in adult patients with acute hypoxemic respiratory failure: a meta-analysis and systematic review. *Journal of Critical Care*.

[32] Mauri T., Alban L., Turrini C. (2017). Optimum support by high-flow nasal cannula in acute hypoxemic respiratory failure: effects of increasing flow rates. *Intensive Care Medicine*.

[33] Ritchie J. E., Williams A. B., Gerard C., Hockey H. (2011). Evaluation of a humidified nasal high-flow oxygen system, using oxygraphy, capnography and measurement of upper airway pressures. *Anaesthesia and Intensive Care*.

[34] Austin M. A., Wills K. E., Blizzard L., Walters E. H., Wood-Baker R. (2010). Effect of high flow oxygen on mortality in chronic obstructive pulmonary disease patients in prehospital setting: randomised controlled trial. *BMJ*.

[35] Singh D., Agusti A., Anzueto A. (2019). Global strategy for the diagnosis, management, and prevention of chronic obstructive lung disease: the GOLD science committee report 2019. *European Respiratory Journal*.

[36] Bräunlich J., Seyfarth H. J., Wirtz H. (2015). Nasal high-flow versus non-invasive ventilation in stable hypercapnic COPD: a preliminary report. *Multidisciplinary Respiratory Medicine*.

[37] Chatila W., Nugent T., Vance G., Gaughan J., Criner G. J. (2004). The effects of high-flow vs. low-flow oxygen on exercise in advanced obstructive airways disease. *Chest*.

[38] Kang B. J., Koh Y., Lim C. M. (2015). Failure of high-flow nasal cannula therapy may delay intubation and increase mortality. *Intensive Care Medicine*.

[39] Messika J., Ben Ahmed K., Gaudry S. (2015). Use of high-flow nasal cannula oxygen therapy in subjects with ARDS: a 1-year observational study. *Respiratory Care*.

