# Supplementary figures and images for: Use High-Flow Nasal Cannula for Acute Respiratory Failure Patients in the Emergency Department: A Meta-Analysis Study
Source: Emerg Med Int. 2019 Oct 13;2019:2130935. doi: 10.1155/2019/2130935 (PMC6815584; doi:10.1155/2019/2130935)

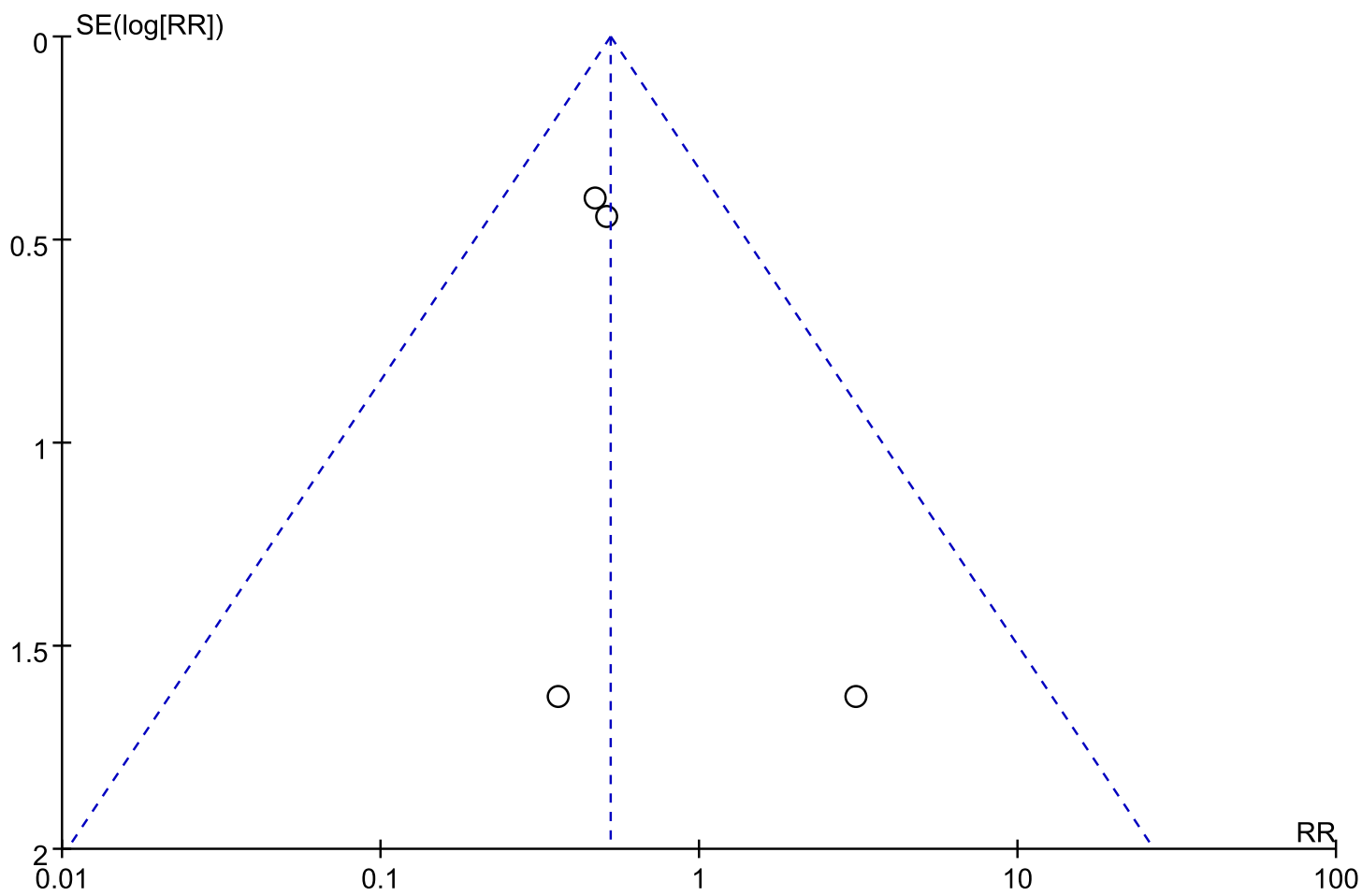

Supplement: Supplementary Materials — A visual inspection of the funnel plot revealed no publication bias. [file 2130935.f1.pdf]
